# LiNbO_3_ Thin Films through a Sol–Gel/Spin-Coating Approach Using a Novel Heterobimetallic Lithium–Niobium Precursor

**DOI:** 10.3390/nano14040345

**Published:** 2024-02-11

**Authors:** Francesca Lo Presti, Anna Lucia Pellegrino, Quentin Micard, Guglielmo Guido Condorelli, Samuel Margueron, Ausrine Bartasyte, Graziella Malandrino

**Affiliations:** 1 Dipartimento di Scienze Chimiche, Università degli Studi di Catania, and INSTM UdR Catania, Viale Andrea Doria 6, I-95125 Catania, Italy; francesca.lopresti@unict.it (F.L.P.); annalucia.pellegrino@unict.it (A.L.P.); guido.condorelli@unict.it (G.G.C.); 2 FEMTO-ST Institute, University of Franche-Comté, ENSMM CNRS UMR 6174, 26 Rue de l’Epitaphe, F-25030 Besançon, Francesamuel.margueron@femto-st.fr (S.M.); ausrine.bartasyte@femto-st.fr (A.B.); 3 Institut Universitaire de France, 1 rue Descartes, F-75231 Paris, France

**Keywords:** LiNbO_3_ porous films, sol–gel deposition, X-ray diffraction, lithium ion batteries

## Abstract

Lithium niobate is a lead-free material which has attracted considerable attention due to its excellent optical, piezoelectric, and ferroelectric properties. This research is devoted to the synthesis through an innovative sol–gel/spin-coating approach of polycrystalline LiNbO_3_ films on Si substrates. A novel single-source hetero-bimetallic precursor containing lithium and niobium was synthesized and applied to the sol–gel synthesis. The structural, compositional, and thermal characteristics of the precursor have been tested through attenuated total reflection, X-ray photoelectron spectroscopy, thermogravimetric analysis, and differential scanning calorimetry. The LiNbO_3_ films have been characterized from a structural point of view with combined X-ray diffraction and Raman spectroscopy. Field-emission scanning electron microscopy, energy dispersive X-ray analysis, and X-ray photoelectron spectroscopy have been used to study the morphological and compositional properties of the deposited films.

## 1. Introduction

The broad range of applications offered by ferroelectric and piezoelectric materials has generated significant interest in sensors, actuators, memories, and acoustic and optical devices. Recently, such increased attention can be attributed to the rising demand for exceptional materials in diverse fields, including microelectronics, integrated photonic circuits, surface acoustics, and catalysis. Lithium niobate (LiNbO_3_—LN), among several other alternatives, has emerged as a crucial component in the advancement of optical and acoustic technologies for its excellent pyroelectric, ferroelectric, nonlinear optical, electro-optical, and piezoelectric properties [1,2]. When lithium niobate is in its ferroelectric state (below its Curie temperature of 1483 K), its structure is composed of oxygen atoms arranged in a distorted hexagonal closed packed configuration. In the structure, the octahedral holes are alternatively filled by Li atoms, Nb atoms, or vacancies. The cations are slightly off-center along the z-axis within the oxygen cages or within an oxygen plane rather than precisely centered, resulting in the spontaneous polarization of LiNbO_3_ along this same axis [3,4]. LN has played a crucial role in the development of non-linear optics and electro-optic devices as a bulk single crystal [5,6]. The molar ratio of Li to Nb in the LN phase, the presence of impurities, and the quantity of vacancies and antisites in the cation sublattice are the main factors affecting the physical and structural properties of LiNbO_3_ [7]. In fact, the congruent composition of LN is nonstoichiometric (48.38 mol% Li_2_O) [8]. Therefore, a more elaborate growth process is required for the development of a stoichiometric single crystal of LN than the well-known Czochralski process used for congruent LN single crystal growth [9,10]. The potential for developing LiNbO_3_ thin films is of particular interest for integrated and miniaturized devices such as optical filters, optical waveguides or modulators, and reduced control voltage [6,11,12]. In fact, epitaxial thin films of LN have found applications in various devices so far, including optical switches [13], photonic crystals [14], solid-state batteries [15], surface acoustic wave (SAW) and bulk acoustic wave (BAW) devices [16,17,18], and quantum memories [19]. In addition, it has been reported that incorporating LiNbO_3_ layers into lithium ion battery (LIB) systems might enhance their stability, mitigate unwanted reactions, and facilitate the transfer of lithium across surfaces [20,21]. Thus, the research on LIBs has been primarily focused on defects and nanoscale entities, including mesoporous materials, nanoparticles, and nanoplates [22,23,24,25]. Therefore, while optical technologies have been known to use LiNbO_3_ single crystals or epitaxial films, polycrystalline or amorphous-based LiNbO_3_ layers are needed for applications in LIBs, being positioned at the electrode/electrolyte interface to optimize the interface characteristics of solid-state LIBs for industrial applications. 

Another added value of the LiNbO_3_ phase is that, together with other materials such as (K,Na)NbO_3_ [26,27] and BiFeO_3_ [28,29] phases, it is a lead-free material [30]. The significant attention garnered by lead-free (K,Na)NbO_3_ arises from its exceptional ferroelectric, dielectric, and piezoelectric features, which are comparable to most lead-based piezoelectric materials [31,32]. In addition, together with its remarkable transparency in the visible spectrum and luminescence potential, (K,Na)NbO_3_ is a promising material for use in light emitters, optoelectronics, and opto-electronic sensors [33]. On the other hand, the inorganic BiFeO_3_ perovskite is widely known for its multiferroic properties, possessing both ferroelectric and antiferromagnetic characteristics [34,35]. Apart from these two features, BiFeO_3_ has attracted attention because of its outstanding properties such as strong magnetoelectric coupling, high spontaneous polarization, and high Curie temperature [36,37]. In addition, the piezo-enhanced photocatalytic activity of BiFeO_3_ based composites has recently been shown [38]. These properties imply that there is a great deal of potential for several uses. 

Concerning the methods employed thus far to deposit thin films of LiNbO_3_, the most commonly utilized techniques include liquid phase epitaxy (LPE) [39], chemical beam vapor deposition [40], metalorganic chemical vapor deposition (MOCVD) [16,41,42,43,44], pulsed layer deposition (PLD) [45,46], molecular beam epitaxy (MBE) [47,48], RF sputtering [49,50,51], atomic layer deposition (ALD) [52,53], and sol–gel deposition [54,55]. In the early 1990s, Nashimoto et al. reported the sol–gel deposition of epitaxial LN films from lithium ethoxide and niobium pentaethoxide with varying water contents on sapphire substrates [56]. They discovered that films made from solutions that had not been pre-hydrolyzed showed epitaxial growth on sapphire, but adding water to promote the sol hydrolysis favored the creation of a random microstructure [56]. Other research groups have recently proposed a promising method for the deposition of high-quality LiNbO_3_ using a combination of Li_2_CO_3_, Nb_2_O_5_, ethylene glycol, and citric acid and employing spin-coating to deposit the LiNbO_3_ nanostructures [57]. Structural data revealed that the nanostructured LN exhibited a polycrystalline nature, resulting in poor optical characteristics [57]. Actually, the sol–gel deposition method offers a number of significant advantages, such as the capacity to operate at low temperatures, the ability to precisely manage composition at the molecular level, and a guarantee of atomic homogeneity and purity of the final product. Indeed, the selection of appropriate molecular precursors is crucial in the sol–gel synthesis, particularly for multi-component systems like LiNbO_3_ [58]. To create sols with a high degree of uniformity, it is essential to select the right molecular precursors. This selection ensures that clusters of molecules in the solution cross-link favoring the process of gelation, while avoiding unfavorable processes such as precipitation [59]. The metal alkoxides of Nb(OC_2_H_5_)_5_ and LiOC_2_H_5_ have been the most frequently employed precursors for the deposition of LiNbO_3_ thin films [60,61].

Very few lithium precursors are known for LN deposition techniques using the vapor phase, such as MOCVD or ALD, or from solutions, such as sol–gel deposition. The vapor pressure of known organometallic Li precursors is typically low. Research on the coordination of new β-diketonate Li precursors with polyether has been recently published [62]. Engineering the coordination sphere of Li^+^ ions has led to the synthesis of intriguing compounds with desirable functional characteristics for use in LiNbO_3_ deposition approaches. Preliminary deposition studies have been applied to the fabrication of epitaxial LiNbO_3_ films on sapphire substrates through a pulsed injection MOCVD reactor utilizing [Li_12_(hfa)_12_monoglyme●4H_2_O]_n_ and Nb(tmhd)_4_ in a 2:1 Li/Nb ratio [62].

In the production of thin films composed of multiple elements, it would be a great advantage to employ a single precursor that incorporates all the necessary elements in the desired stoichiometry for the growth of the final product. Recently, sodium and rare earth (RE) heterobimetallic complexes have been synthesized and used to deposit NaREF_4_ films using chemical vapor deposition [63]. These “third-generation” precursors have proven to be volatile, thermally stable, and capable of concurrently supplying every element required for the desired phase.

Thus, producing a single bimetallic precursor that contains all necessary components (Li, Nb, and O) would ensure better monitoring of all process variables and greater atomic uniformity, reduce the occurrence of undesirable contaminants, and improve control over the production process. In fact, the use of LN thin films is still severely constrained by the challenge of regulating the mix of components. 

To ensure the stoichiometric composition of LiNbO_3_ films, we here present an extensive investigation on the synthesis of a multi-metal precursor that combines Li and Nb elements in the same molecule. This innovative single hetero-bimetallic precursor has the formula “Li_2_Nb(hfa)_7_•diglyme•xH_2_O” and simultaneously supplies all the elements required for the target phase, namely, two metal ions and oxygen. The structural, compositional, and thermal characteristics of “Li_2_Nb(hfa)_7_•diglyme•xH_2_O” have been tested through attenuated total reflection Fourier transform infrared spectroscopy (ATR-FTIR), energy dispersive X-ray analysis (EDX), X-ray photoelectron spectroscopy (XPS), thermogravimetric analysis (TGA), and differential scanning calorimetry (DSC). Subsequently, a unique sol–gel/spin-coating method has been used to produce polycrystalline LN thin films, using this innovative heterobimetallic fluorinated β-diketonate precursor, for potential applications in lithium ion batteries [20]. 

The polycrystalline LiNbO_3_ thin films have been characterized from a structural point of view with a combined XRD/Raman study and from morphological and compositional points of view with field-emission scanning electron microscope (FE-SEM), energy dispersive X-ray (EDX) analysis, and X-ray photoelectron spectroscopy.

## 2. Materials and Methods

### 2.1. Starting Materials

Lithium hydroxide (LiOH), niobium pentachloride (NbCl_5_), and 1,1,1,5,5,5-hexafluoro-2,4-pentanedione (H-hfa) were purchased from Strem Chemicals Inc. Bischheim (France) and were used without other purifications. Diglyme [bis(2-methoxyethyl)ether] and dichloromethane (CH_2_Cl_2_) were purchased from Sigma Aldrich (Merck KGaA, Darmstadt, Germany).

### 2.2. Synthesis of “Li_2_Nb(hfa)_7_•diglyme•xH_2_O”

The NbCl_5_ (0.312 g, 1.2 mmol) was first suspended in dichloromethane (40 mL). An excess of LiOH (0.771 g, 12.4 mmol) and diglyme (0.158 g, 1.2 mmol) were added to the suspension. Hhfa (1.74 g, 8.4 mmol) was added after 10 min, and the mixture was refluxed under stirring conditions for 1 h. The excess of LiOH was filtered out. After the solvent partially evaporated, the light-yellow colorless crystals formed. The crystals were collected, cleaned with pentane, filtered, and dried in air. The reaction yield was 66%. The melting point of the product was 69–78 °C. 

### 2.3. Characterization of the Precursor

The attenuated total reflection infrared spectrum was recorded using a Spectrum Two FT-IR PerkinElmer spectrometer (PerkinElmer, Milano, Italy). Thermogravimetric analyses were conducted using Mettler Toledo TGA2 (Mettler-Toledo S.p.A., Milano, Italy) and STAR^e^ software. Dynamic thermal studies were performed under 50 sccm pure nitrogen flow conditions, at atmospheric pressure, and with a heating rate of 5 °C/min. The sample weight was around 10 mg. Differential scanning calorimetry analyses were carried out using a Mettler Toledo Star System DSC 3 (Mettler-Toledo S.p.A., Milano, Italy) under purified nitrogen flow conditions (50 sccm) at atmospheric pressure with a 5 °C/min heating rate. X-ray photoelectron spectroscopy (XPS) was carried out using a PHI 5600 multi-technique ESCA Auger spectrometer (Physical electronics Inc., Chanhassen, MN, USA) equipped with a standard Mg-K_α_ X-ray source. The XPS binding energy (BE) scale was calibrated using the C 1 s peak of adventitious carbon at 285.0 eV.

### 2.4. Sol–Gel Deposition

The sol–gel reaction occurred in the ethanol/water solution of “Li_2_Nb(hfa)_7_•diglyme•xH_2_O” single precursor. For the sol hydrolysis, the following molar ratio of precursor, solvent and catalyst was used: “Li_2_Nb(hfa)_7_•diglyme•xH_2_O”/87 C_2_H_5_OH/3H_2_O/0.8 CF_3_COOH. The sol was spin-coated on 1 cm × 1 cm Si (100) substrates after stirring it at 60 °C for 20 h. A SPIN-150 spin-coater (SPS Europe, Putten, The Netherlands) was used to perform the spin-coating operation with an acceleration of 1000 RPM/sec and a speed of 3000 RPM (revolutions per minute). In more detail, the sample was heated in air for 10 min at 350 °C after each 60 s spin of 0.2 mL of the gel on the substrate. A final annealing procedure at temperatures between 600 °C and 800 °C was carried out for one hour in air. A computer-controlled hardware setup and K-type thermocouples with a ±1 °C precision was used to monitor the temperature during the whole heating procedure.

### 2.5. Film Characterization

A Smartlab Rigaku diffractometer (Rigaku, Tokyo, Japan) with a Cu K_α_ radiation rotating anode at 45 kV and 200 mA was used for structural analysis. The patterns were recorded in grazing incident mode (0.5°) with a 0.02° step resolution across a range of 15 to 60 degrees. Film morphology was observed by field-emission scanning electron microscopy using a ZEISS SUPRA VP 55 microscope (ZEISS, Jena, Germany). The EDX spectra were recorded using an INCA-Oxford detector (Oxford Instruments, Abingdon, UK), having a resolution of 127 eV as the FWHM of the Mn K_α_. Non-polarized Raman measurements were collected by using an S&I MonoVista Raman spectrometer (Spectroscopy & Imaging GmbH, Warstein, Germany) with excitation at 532 nm using an objective with ×100 magnification, resulting in an analyzed area with a diameter of 1 µm. For comparison purposes, stoichiometric LiNbO_3_ powder was prepared using Li_2_CO_3_ (99.9%) and Nb_2_O_5_ (99.9%) with molar ratios of 50% as starting chemicals. To eliminate moisture, the Li_2_CO_3_ was dried for 16 h at 250 °C. The powders were mixed, milled, and sintered for 120 h at 1000 °C. XPS characterization was carried out using the same instrument and conditions reported in the section of precursor characterization.

## 3. Results and Discussion

### 3.1. Heterobimetallic Li-Nb Single-Source

In order to achieve the precise stoichiometric balance in LiNbO_3_ films, we have effectively synthesized a multi-metal precursor by combining Li and Nb elements within a single molecule. The resulting innovative single hetero-bimetallic precursor, having the formula “Li_2_Nb(hfa)_7_•diglyme•H_2_O”, represents the first case of a Li-Nb metalorganic compound. This novel type of precursor provides all the essential metal elements required for the desired phase while maintaining suited properties for their application in the fabrication of LiNbO_3_ films. The formation of this multi-metal complex was achieved through a one-pot reaction, where LiOH, NbCl_5_, hexafluoroacetylacetone, and diglyme were mixed in dichloromethane. A LiOH/NbCl_5_/Hhfa/diglyme stoichiometry of 7:1:7:1 was used following the reaction:

$$\;7\;\mathrm{LiOH}+{\mathrm{NbCl}}_{5}+7\;\mathrm{Hhfa}+\mathrm{diglyme}\to \left[{\mathrm{Li}}_{2}\mathrm{Nb}{\left(\mathrm{hfa}\right)}_{7}•\mathrm{diglyme}•{\mathrm{xH}}_{2}O\right]+5\mathrm{LiCl}+H_{2}O$$

To determine the structural nature of the adduct, numerous attempts were made to grow single crystals, but unfortunately all the single crystals were twinned. Thus, the complex nature has been investigated through FT-IR spectroscopy, XPS, DSC analysis, and melting point measurements. The 2:1 Li/Nb ratio of the precursor determined through XPS (vide infra), though different from that present in the LiNbO_3_ phase, is actually an added value considering that usually a nominal composition of the 2Li/1Nb ratio is needed in order to produce stoichiometric LiNbO_3_ phase films, due to the volatility of the Li_2_O phase.

**Figure 1:**
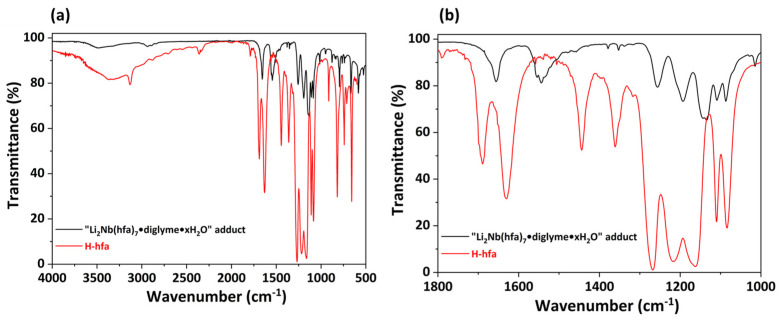
Comparison between the ATR/FT-IR spectrum of the “[Li_2_Nb(hfa)_7_•diglyme•xH_2_O]” adduct (black line) and the Hhfa neat sample (red line) (**a**) and an enlarged region between 1000 cm^−1^ and 1800 cm^−1^ (**b**).

The transmittance spectrum obtained from ATR-FTIR analysis of the complex, as depicted in Figure 1, reveals the presence of a small peak around 3600 cm^−1^ [64]. This peak is indicative of stretching vibrations related to --OH groups, implying the presence of a small number of water molecules within the coordination sphere of the complex. In the adduct, the presence of peaks at 1656 and 1544 cm^−1^ corresponds to the stretching vibrations of C=O and C=C bonds, respectively [62]. These peaks are characteristic of the β-diketonate framework, and the peak shifts compared to the position of the free ligand (C=O and C=C bonds at 1690 and 1630 cm^−1^, respectively) confirm the coordination of the hfa ligand, indicating the complex formation. The peaks detected in the range of 1000−1250 cm^−1^ exhibit characteristics typical of polyether C−O−C bending and/or stretching, which are combined with the features of C−F stretching of the anionic ligand [64,65].

**Figure 2:**
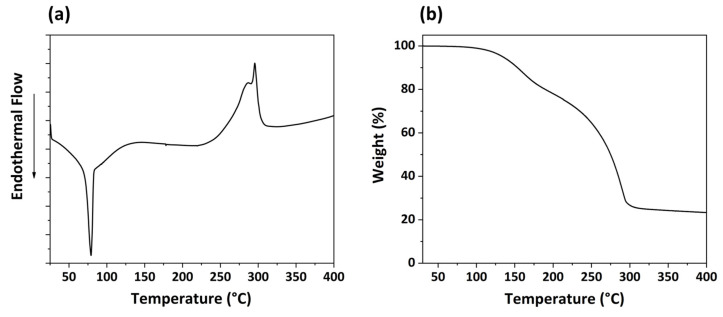
DSC (**a**) and TGA (**b**) curves of the “[Li_2_Nb(hfa)_7_•diglyme•xH_2_O]” adduct.

Investigating the thermal properties of the adduct is an essential aspect in ensuring its effective application in vapor or solution processes. To this end, differential scanning calorimetry (DSC) analysis and thermogravimetric (TG) measurements were conducted under atmospheric pressure, using purified nitrogen as flowing gas. Figure 2a illustrates the DSC curve for the “[Li_2_Nb(hfa)_7_•diglyme•xH_2_O]” sample. The adduct demonstrates a well-defined melting point at 79.2 °C, which aligns closely with the melting point observed using a Kofler microscope. Following the melting, the precursor initiates decomposition within the temperature range of 285–295 °C, as supported by the presence of an exothermic band within this range. The TG curve shown in Figure 2b displays two distinct weight loss steps. The first step, resulting in a 20% mass loss, occurs within the temperature range of 105 °C to 180 °C. The second weight loss step, occurring between 240 °C and 295 °C, is primarily attributed to the decomposition of the precursor, resulting in a residue of 24%. According to this finding, such a heterobimetallic precursor does not possess good mass-transport properties for the conventional MOCVD approach, but it is expected to work well for the deposition of LiNbO_3_ thin films through solution processes, either sol–gel or solution-assisted MOCVD.

**Figure 3:**
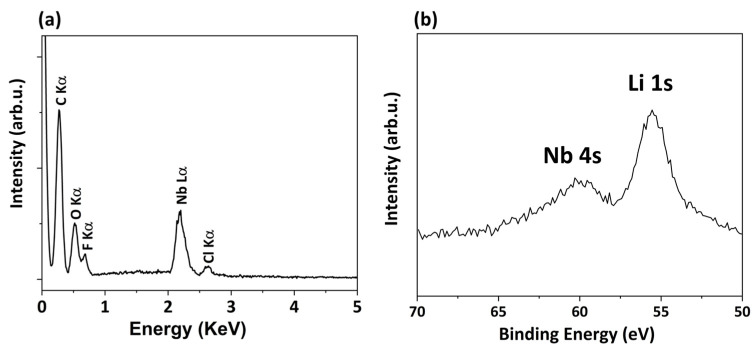
EDX spectrum (**a**) and XPS Li-Nb binding energy (**b**) of the “[Li_2_Nb(hfa)_7_•diglyme•xH_2_O]” adduct.

Subsequently, the complex powders have been characterized by means of EDX and XPS techniques to accurately evaluate the presence and quantification of the different elements within the structure. Figure 3a shows the EDX spectrum, which reveals the presence of the Nb L_α_ peak at around 2.2 KeV. The F K_α_, O K_α_, and C K_α_ elements are also confirmed by the presence of peaks at 0.69 KeV, 0.52 KeV, and 0.28 KeV, respectively, due to the hfa and diglyme ligand. A small peak at 2.6 keV is due to the K_α_ peak of Cl, present as an impurity arising from the NbCl_5_ reagent. It should be emphasized that special consideration was not paid to their quantitative analysis, since the quantification of light elements is highly critical, especially in this case of an insulating powder sample. 

Moreover, given the lightweight nature of lithium, it becomes essential to employ supplementary characterization techniques like XPS analysis to gain comprehensive insights into the nature of the complex under investigation. The spectral region shown in Figure 3b indicates the presence of Nb 4s at 60.1 eV and Li 1s at 55.5 eV. 

The chemical composition obtained from the XPS analysis is presented in Table 1. Based on the quantitative analysis, it is evident that both lithium and niobium are present in the precursor structure. The ratio of these two elements is approximately 2Li/1Nb. This ratio implies the existence of an adduct with twice as much lithium as niobium. The sample survey spectrum is reported in Figure S1a, while the binding energy regions of all other elements forming the precursor’s structure, i.e., Nb 3d, O1s, C1s, Li 1s, and F1s, are reported in Figure S1b.

### 3.2. Sol–Gel Synthesis of LiNbO_3_ Films from the Single Li-Nb Precursor

The “[Li_2_Nb(hfa)_7_•diglyme•xH_2_O]” heterobimetallic adduct has been confirmed to be a reliable single source of Li and Nb elements by using it in the sol–gel synthesis of lithium niobate films on silicon substrates. The stoichiometric ratio of 2Li/1Nb, though different from that observed in the LiNbO_3_ phase, is actually an advantage since usually a higher amount of lithium precursor is needed to produce the LN films with the correct ratio, considering that usually a more lithium-rich mixture is used in the pulsed injection metalorganic vapor phase deposition to produce single-phase LN films due to the lithium oxide volatility. Each experiment has been duplicated two or three times to guarantee the consistency and reproducibility of the methodology. In this particular process, ethanol has been used as the solvent to dissolve the precursor, while trifluoroacetic acid (CF_3_COOH) has been employed as the catalyst for sol hydrolysis. The molar ratios of the precursors, solvent, and catalyst are provided below:

“[Li_2_Nb(hfa)_7_•diglyme•xH_2_O]”/87 Et-OH/3 H_2_O/0.8 CF_3_COOH

**Scheme 1:**
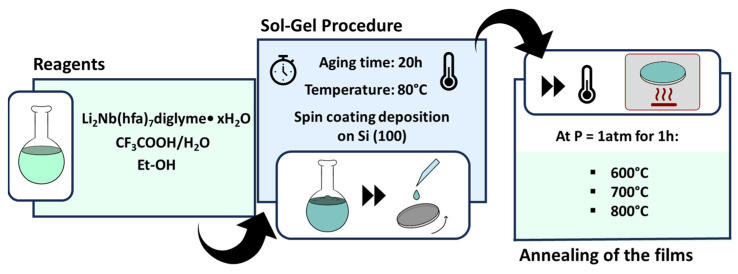
Scheme of the sol–gel process of LiNbO_3_ films.

After being aged at 60 °C for 20 h, the sol has been spun onto Si (100) substrates. According to published studies, annealing temperatures ranging from 600 °C to 800 °C have been investigated (see Scheme 1) [14,66].

**Figure 4:**
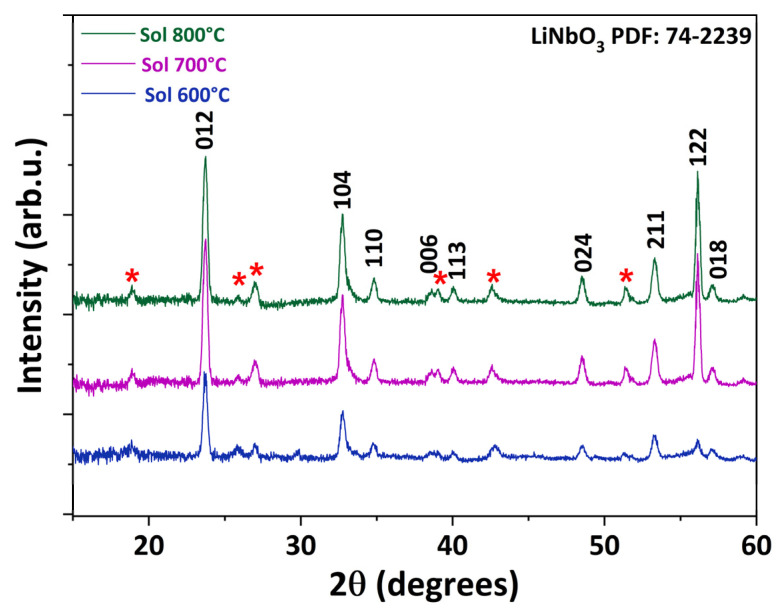
GIXRD patterns of LN thin film annealed at 600–800 °C. Red stars indicate peaks of an impurity phase.

The grazing incidence X-ray diffraction (GIXRD) patterns, depicted in Figure 4, illustrate the variations in LiNbO_3_ thin films formed on silicon substrates through annealing at different temperatures. Indeed, the analysis of diffraction patterns confirms the formation of polycrystalline LiNbO_3_ phase films across the temperature range of 600–800 °C, with an increase in crystallization starting between 600 and 700 °C, as confirmed through Raman measurements. These patterns exhibit distinct diffraction peaks at specific 2θ values, including 23.72°, 32.74°, 34.84°, 38.98°, 40.04°, 48.5°, 53.3°, 56.12°, and 57.06°. These peaks correspond to the characteristic reflections of the LiNbO_3_ trigonal phase, specifically the 012, 104, 110, 006, 113, 024, 116, 122, and 018 reflections, respectively, as documented in the ICDD database (PDF no. 074-2239). The relative intensities of the peaks observed in these patterns are well aligned with the reported values of the ICDD database, suggesting that there is no preferential direction of growth. Unfortunately, there are a few extra peaks at 19.70°, 25.60°, 26.70°, 39.22°, 43.52°, and 52.40°, visible in the patterns and indicated with red stars, which are related to an impurity phase likely due to Li and/or Nb containing systems. 

**Figure 5:**
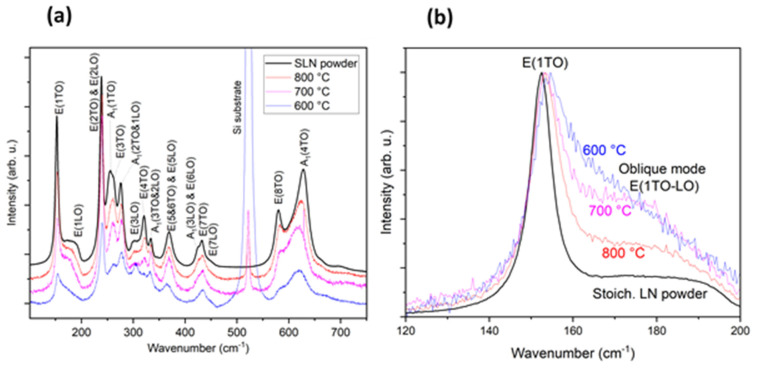
(**a**) Comparison of Raman spectra and (**b**) E(1TO) mode profiles with normalized intensity of LN thin film annealed at 600–800 °C.

To gain further insights, additional characterizations were conducted. Specifically, a Raman study was performed on the samples deposited on silicon substrates and annealed at 600 °C, 700 °C, and 800 °C. It is known that the LiNbO_3_ crystal belongs to the R3c space group (Z = 2) at room temperature with 4A_1_ + 9E + 5A_2_ optical modes. Only the A_1_ and E modes are Raman-active [67]. We investigated how different annealing temperatures influenced the Raman spectra, as presented in Figure 5a. The Raman spectra of synthesized polycrystalline films were compared to that of stoichiometric LN powder Raman modes and identified according to the assignment given in Ref. [67]. Notably, the phonon modes observed in the film spectra closely resemble the equivalent modes observed for stoichiometric LiNbO_3_ powders. No additional modes were observed, indicating that the film is composed mainly of a single LiNbO_3_ phase and that the additional impurity phase, observed in XRD patterns, could be segregated locally [68]. In fact, while micro-Raman spectroscopy is a punctual analysis with a much smaller analyzed area, the XRD measurement in grazing incidence analyzes a large area of the films. The wavenumbers of observed modes with highest intensity were compared to those reported for the single crystals (Table 2). It is important to note that fundamental Raman modes are present together with quasi-/oblique modes due to angular dispersion of crystallite orientation in LN polycrystalline samples [69]. This introduces the mode wavenumber deviation from those observed in the single crystal samples. Nevertheless, the mode wavenumbers remain relatively close to those measured for single crystals and agree very well with the measured intensity ratios and the wavenumbers of the Raman modes in the stoichiometric LN powder. It is known that the E(6TO) mode is particularly sensitive to the residual stresses in the LN films [70]. The films sintered at 700 °C and 800 °C present the E(6TO) mode with a very close wavenumber to that of stress-free powders. This indicates minimal mechanical stress within the films. One can note that LN films crystallized at 600 °C present less defined Raman mode profiles, indicating lower crystalline quality/higher defect concentration than films annealed at 700–800 °C. The widths/dampings of Raman modes are dependent on the defect concentration, including nonstoichiometry and other crystalline defects in the LN structure [67,70]. Therefore, E(1TO) mode profiles of sintered films at different temperatures were compared to this mode profile in powders sintered at 1000 °C (Figure 5b). The increase in the annealing temperature considerably ameliorates the crystalline quality of the films. We would like to stress that the broader Raman modes can also be related to the deviation from the stoichiometric Li composition as well as different oblique mode profiles (Figure 5b) related to the different local distribution of crystallite orientation.

The powders normally present larger grains than submicrometer grains in thin films, vide infra, and this results in less pronounced oblique mode dispersion in powders than in the thin films. 

Compositional EDX analysis of the LN films deposited at different temperatures confirmed the presence of a phase free of C or F impurities, since only the peaks corresponding to Nb and O were present at 2.19 KeV and 0.53 KeV, respectively. 

**Figure 6:**
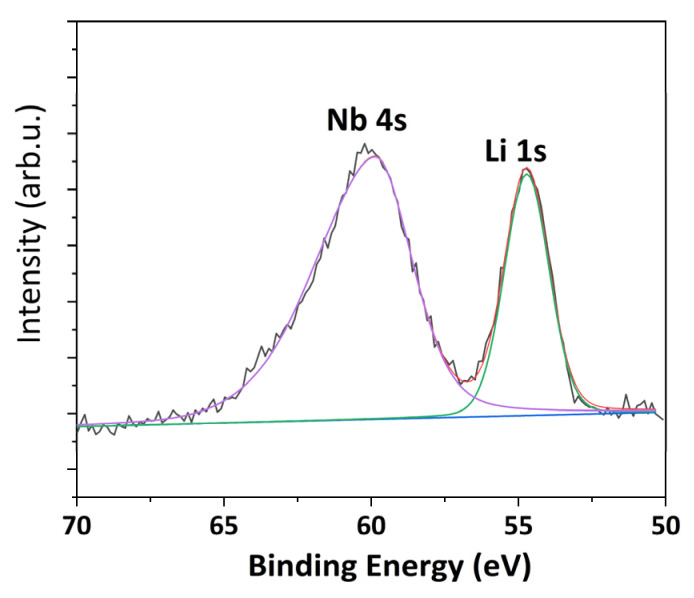
XPS Li-Nb region of a LN film annealed at 700 °C on Si (100): the green line is the Gaussian shape fitting the Li 1s peak and the purple line is the asymmetric Gaussian–-Lorentzian shape fitting the Nb 4s peak.

XPS quantitative analysis confirms a stoichiometric ratio of Li/Nb close to 1:1. The accuracy of XPS in Li concentration was found to be 20% [71]. The presence of C is attributed to adventitious contamination, while the low percentage of F suggests some fluorine contamination from the β-diketonate ligands (as shown in Table 3). In the region reported in Figure 6, we observe the presence of Nb 4s at 60.2 eV and Li 1s at 54.7 eV, in accordance with literature data [40]. For the quantitative evaluation, a Gaussian shape was used for the Li 1s peak, while an asymmetric Gaussian–Lorentzian shape was used for the Nb 4s peak [72,73]. The sample survey spectrum and spectra of additional elements that form the LiNbO_3_ phase are reported in Figure S2a and Figure S2b, respectively.

**Figure 7:**
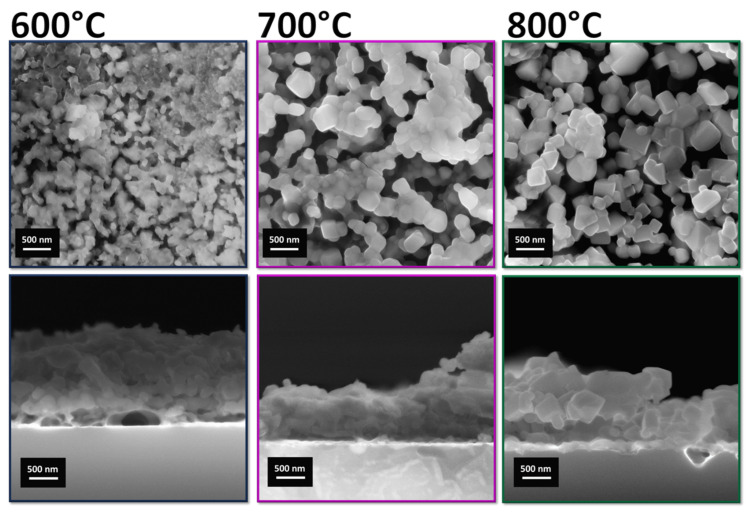
FE-SEM and cross-sectional images of LN films annealed at 600 °C, 700 °C, and 800 °C.

The morphology of the samples deposited at various temperatures has been examined using FE-SEM microscopy, as illustrated in Figure 7. The observed morphology exhibits characteristic features consistent with sol–gel deposition, displaying a highly wrinkled structure accompanied by small-sized grains. However, as the temperature is increased, a significant transformation becomes evident. In fact, upon increasing the temperature from 600 °C to 800 °C, a noticeable increase in crystallinity becomes apparent in the morphology of the LN films deposited on Si substrate: the individual grains become more distinct and well-defined, leading to a crystallinity enhancement in the overall surface structure. Specifically, the grains exhibit a pronounced increase in both granularity and definition: at 600 °C, rounded grains of the order of 100 nm are observed; at 700 °C more defined grains with dimensions around 200–300 nm are found; while at 800 °C, the film shows cubic grains ranging from 200 to 500 nm.

The cross-sectional images provide a comprehensive overview of the films deposited on Si substrates, illustrating the variations in film thickness at different deposition temperatures. Upon analysis, it has been observed that the films treated at 600 °C exhibited a thickness of 1.4 ± 0.03 μm. As the deposition temperature increased to 700 °C, the film thickness decreased to 1.0 ± 0.3 μm. Furthermore, the films deposited at 800 °C demonstrated a thickness of 970 ± 80 nm. This observation establishes a clear dependence of film thickness on the treatment temperature. Notably, an increase in temperature resulted in a slight reduction in film thickness, indicating a trend towards enhanced film compactness. As the temperature rose, the film structure appeared to become more tightly packed, leading to a thinner overall thickness. This correlation between treatment temperature and film thickness highlights the importance of temperature control in achieving desired film properties and characteristics.

### 3.3. Sol–Gel Synthesis of LiNbO_3_ Films from Individual Precursors

As a parallel investigation into the synthesis of LiNbO_3_ films, sol–gel/spin-coating experiments have been conducted using individual precursors for lithium and niobium. Specifically, the [Li_2_(hfa)_2_•diglyme•H_2_O] [62] and Nb(tmhd)_4_ precursors, which have been previously reported in the literature, have been used. This comparison aimed to determine whether similar results could be achieved using these individual, well-established precursors and validate the advantages of the single [Li_2_Nb(hfa)_7_•diglyme•xH_2_O] precursor under investigation. 

**Figure 8:**
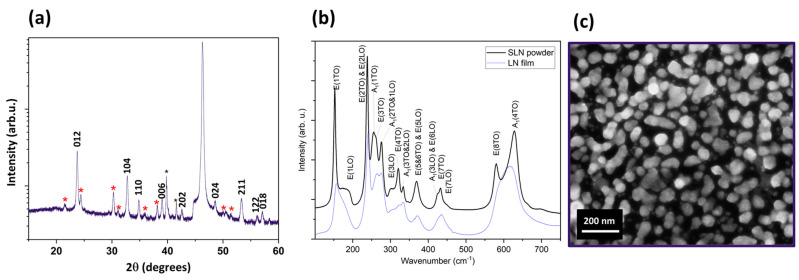
(**a**) GIXRD pattern, (**b**) Raman spectrum, and (**c**) FE-SEM images of a LN thin film obtained from [Li_2_(hfa)_2_•diglyme•H_2_O]/2 Nb(tmhd)_4_ single precursors. Red stars in the XRD pattern indicate peaks of the LiNb_3_O_8_ phase, while black stars are due to the substrate.

Preliminary deposition experiments have been conducted using a sol–gel approach, employing the [Li_2_(hfa)_2_•diglyme•H_2_O] adduct and Nb(tmhd)_4_ dissolved in ethanol solvent in a 1:2 ratio. For the sol hydrolysis process, the molar ratio of the precursor, solvent, and catalyst have been adjusted to 1 [Li_2_(hfa)_2_•diglyme•H_2_O]/2 Nb(tmhd)_4_/87 C_2_H_5_OH/3 H_2_O/0.8 CF_3_COOH. The sol solution has been stirred at 60 °C for 20 h before being spin-coated onto Pt/Si (100) substrates. Subsequently, the spin-coated films have been annealed at 700 °C in air for one hour. Figure 8 depicts the principal structural and morphological characterization of the sample. 

The XRD pattern (Figure 8a) reveals distinct reflections associated with the polycrystalline nature of the LiNbO_3_ phase, alongside some small peaks at 21.60°, 24.60°, 30.25°, 35,80°, and 38.10°, indicated with red stars that could be attributed to the LiNb_3_O_8_ phase (ICDD PDF no. 075-2154). Raman spectroscopy analysis conducted on the sample (Figure 8b) confirms the presence of the LiNbO_3_ phase as well, as evidenced by the presence of characteristic modes associated with this phase. The shift of Raman modes related to the strong presence of the quasi-/oblique modes are due to the local texture of the grains.

The FE-SEM image (Figure 8c) shows the formation of a homogeneous surface, characterized by noticeably smaller and distorted grains compared to those obtained using the previous precursor. This observation suggests that the new single precursor “[Li_2_Nb(hfa) _7_•diglyme•xH_2_O]” may have good potential as a single source for the deposition of LiNbO_3_ thin films.

## 4. Conclusions

Polycrystalline LiNbO_3_ phase films have been synthesized through a sol–gel/spin-coating approach for application in lithium ion batteries. To conveniently develop this material in the form of thin films through solution approaches, the starting point is the engineering of the initial metalorganic precursors. The research has included the synthesis of a novel heterobimetallic precursor, “[Li_2_Nb(Hfa)_7_•diglyme•xH_2_O]”. This innovative precursor offers all the necessary elements for achieving the desired phase. The heterobimetallic adduct has been proved to be a viable single source of Li and Nb elements through use in the sol–gel production of lithium niobate films on silicon substrates. The formation of this complex involving multiple metals has been performed via a one-pot reaction, in which all the metal reagents, the hexafluoroacetylacetone, and diglyme were combined in dichloromethane. Thin films of LiNbO_3_ were developed using a tailored sol–gel approach at different temperatures (600 °C to 800 °C), as confirmed by the GIXRD patterns and Raman spectroscopy. The observed morphology displays features that are typical of sol–gel deposition, including a highly porous structure and small-sized grains. The optimized process has been also compared to the sol–gel/spin-coating synthesis of the LN films using a combination of two separate precursors, the [Li_2_(hfa)_2_•diglyme•H_2_O] and Nb(tmhd)_4_. The effectiveness of the single “[Li_2_Nb(hfa)_7_•diglyme•xH_2_O]” precursor approach, with respect to the use of individual ones, has been proved by the good quality of the prepared polycrystalline LiNbO_3_ films, whose highly porous morphology with small-sized grains makes them appealing and promising for application in lithium ion batteries.

## Figures and Tables

**Table 1:** XPS quantitative analysis of the “[Li_2_Nb(hfa)_7_•diglyme•xH_2_O]” adduct.

XPS Quantitative Analysis					
Li 1s	Nb 3d	C 1s	F 1s	O 1s	Cl 2p
4.3	2.1	32.5	42.0	18.9	0.1

**Table 2:** Comparison of the Raman modes of the sol–gel grown LN films and literature data.

Raman Modes		
E Modes	Our Work (cm^−1^)	Ref. [67] (cm^−1^)
1TO	153	155
2TO-2LO	239	240–241
3TO	262	265
4TO	322	322
6TO	368	370
7TO	434	433
8TO	583	579
**A1 modes**		
1LO-2TO	277	275–276
2LO-3TO	335	334
4TO	624	632

**Table 3:** Quantitative analysis of LN films.

Element	Atomic Percentage
**C 1s**	15.8%
**O 1s**	49%
**F 1s**	6.6%
**Nb 3d**	11.2%
**Li 1s**	13.3%
**Si 2p**	4.1%

## Data Availability

Research data are available to interested researchers upon request.

## Supplementary Materials

The following supporting information can be downloaded at: https://www.mdpi.com/article/10.3390/nano14040345/s1, Figure S1: XPS survey and binding energy regions of the “Li_2_Nb(hfa)_7_•diglyme•xH_2_O” precursor; Figure S2: XPS survey and binding energy regions of a LN film.
