# Supplementary material for: LiNbO3 Thin Films through a Sol–Gel/Spin-Coating Approach Using a Novel Heterobimetallic Lithium–Niobium Precursor
Source: Nanomaterials (Basel). 2024 Feb 11;14(4):345. doi: 10.3390/nano14040345 (PMC10892834; doi:10.3390/nano14040345)
Supplement: Supplementary file 1 [file nanomaterials-14-00345-s001.zip › nanomaterials-2853186-supplementary.pdf]

SUPPORTING INFORMATION

$LiNbO_3$ Thin Films through a Sol–Gel/Spin-Coating Approach Using a Novel Heterobimetallic Lithium–Niobium Precursor

Francesca Lo Presti [1], Anna Lucia Pellegrino [1], Quentin Micard [2], Guglielmo Guido Condorelli [1], Samuel Margueron [2], Ausrine Bartasyte [2,3] and Graziella Malandrino [1,*]

[1] Dipartimento di Scienze Chimiche, Università degli Studi di Catania, and INSTM UdR Catania, Viale Andrea Doria 6, I-95125 Catania, Italy; francesca.lopresti@unict.it (F.L.P.); annalucia.pellegrino@unict.it (A.L.P.); guido.condorelli@unict.it (G.G.C.)
[2] FEMTO-ST Institute, University of Franche-Comté, ENSMM CNRS UMR 6174, 26 Rue de l'Epitaphe,
F-25030 Besançon, France; samuel.margueron@femto-st.fr (S.M.), ausrine.bartasyte@femto-st.fr (A.B.)
[3] Institut Universitaire de France, 1 rue Descartes, F-75231 Paris, France
* Correspondence: gmalandrino@unict.it; Tel.: +39-095-738-5055

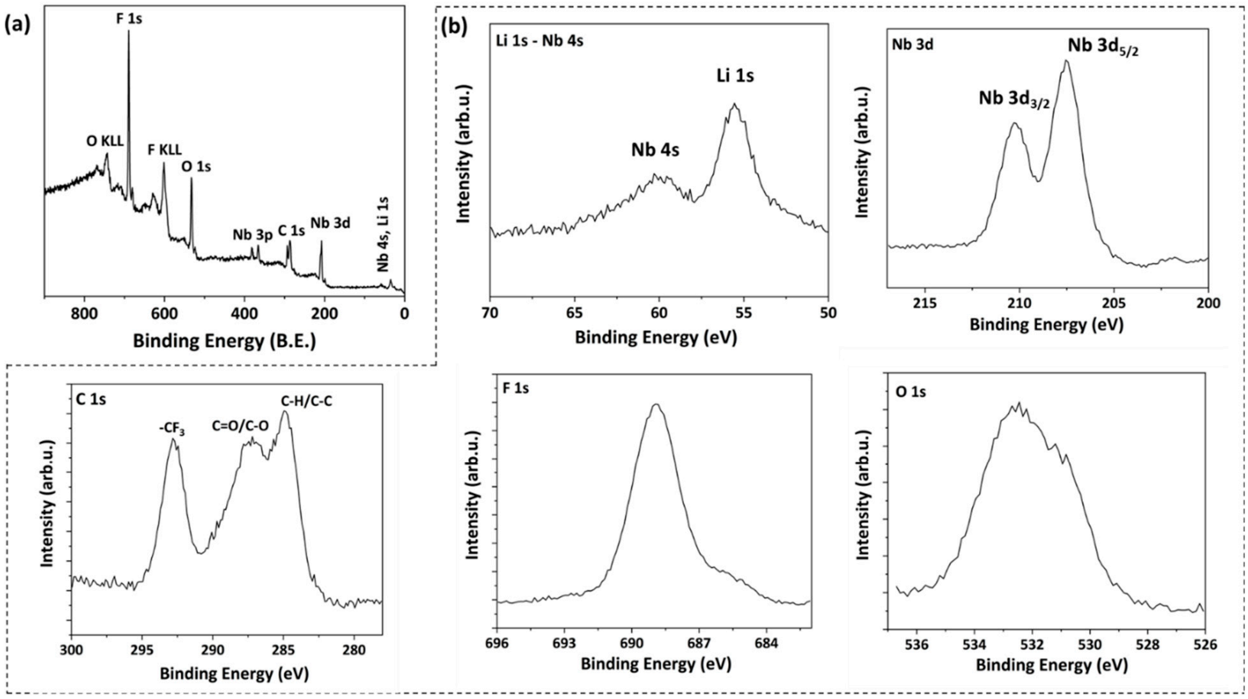

Figure S1. XPS survey (a) and binding energy regions (b) of Li 1s, Nb 4s, Nb 3d, C1s, F 1s, and O1s of the "$Li_2Nb(hfa)_7$•diglyme•xH_2O" precursor.

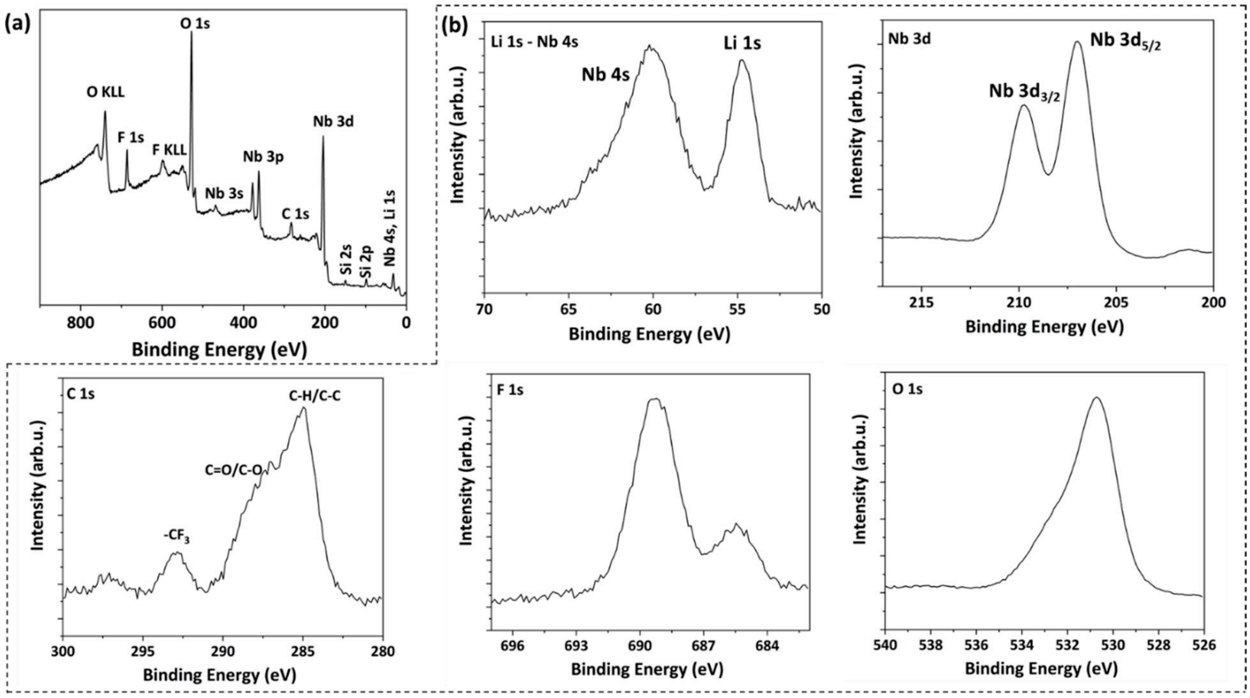

Figure S2. XPS survey (a) and binding energy regions (b) of Li 1s, Nb 4s, Nb 3d, C1s, F 1s, and O1s of a LN film annealed at 700°C on Si (100).
